# Supplementary material for: Silencing DTX3L Inhibits the Progression of Cervical Carcinoma by Regulating PI3K/AKT/mTOR Signaling Pathway
Source: Int J Mol Sci. 2023 Jan 3;24(1):861. doi: 10.3390/ijms24010861 (PMC9821498; doi:10.3390/ijms24010861)
Supplement: Supplementary file 1 [file ijms-24-00861-s001.zip › Figure S1.pdf]

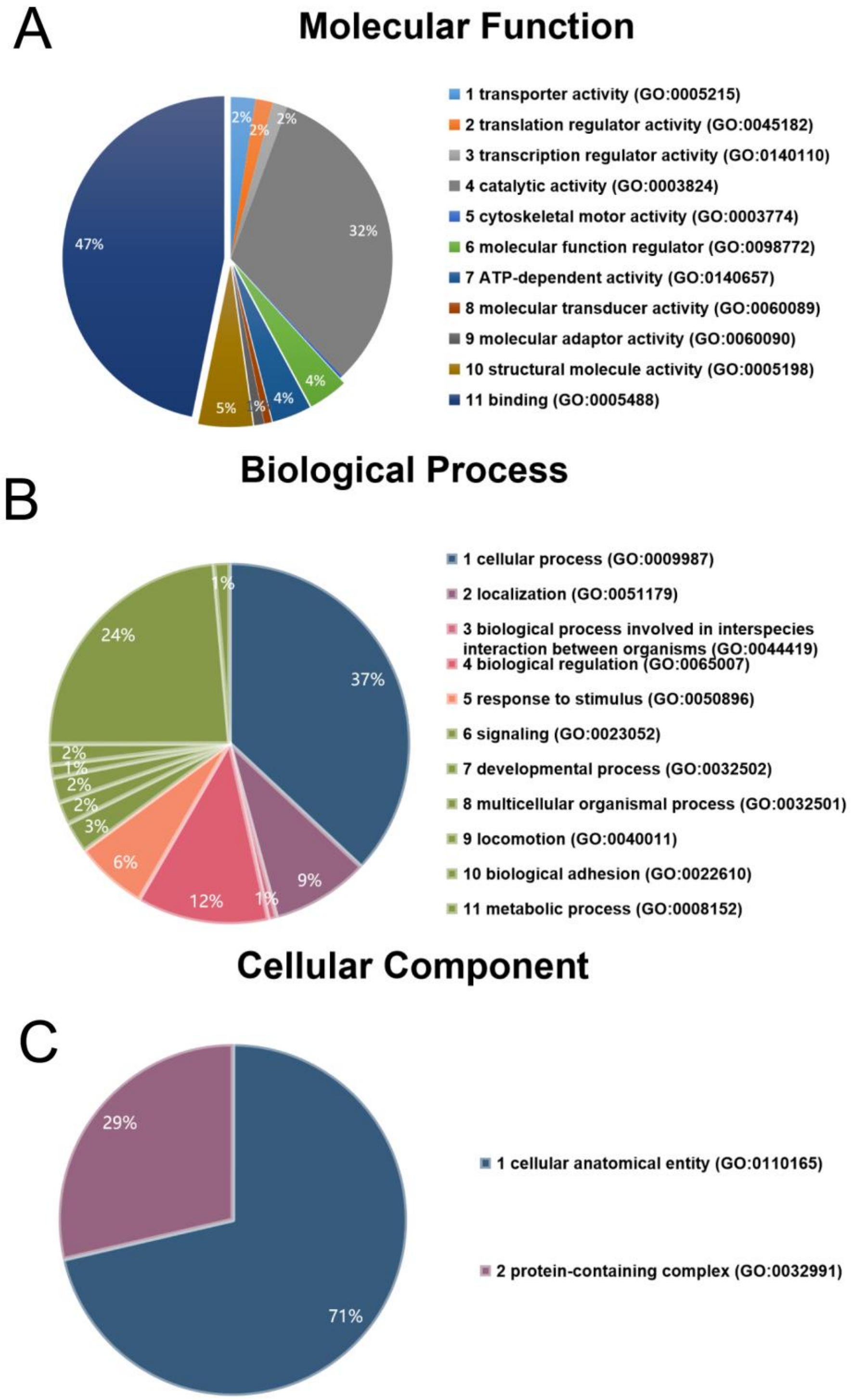


Supplementary Figure S1: Gene ontology categorization of the proteins.

Gene ontology categorization based on the molecular function of the DEPs is shown in Supplementary Figure S1. Most of these proteins were found to be involved in catalytic activity (32%) and binding activities (47%). As for the biological process, 37% proteins were annotated into the cellular process, and 24% were involved in the metabolic process. In terms of cellular component, 71% proteins were involved in cellular anatomical entity and 29% in protein-containing complex.

Figure S1; Gene ontology categorization of the identified proteins based on the molecular function, biological process, and cellular component.
